# News and Notes

**Published:** 1902-10

**Authors:** 


### *NEWS AND NOTES.*

(We are indebted to the *Medical Age* for many of these notes.)

---

CHOLERA has spread from Chinese ports to Japan.

---

AN epidemic of typhus fever is prevailing on the west coast of Ireland.

---

THE marriage of Dr. J. R. Garner and Miss Birdie Patterson, of Atlanta, is announced.

---

THE unveiling of Pasteur's statue took place on August 3, at his birthplace, Dole, Jura.

---

DR. GEORGE BROWN announces that hereafter his practice will be limited to diseases of the nose and throat.

---

DR. CARL GERHARDT, an authority on pediatrics and also on pulmonary diseases, died on his estate of Damberg, Baden, on July 21.

---

SIR FREDERICK TREVES, hitherto one of King Edward's honorary sergeant-surgeons, has been appointed a sergeant-surgeon in ordinary to his majesty.

---

THE Atlanta Society of Medicine has resumed its meetings, suspended during the summer. The meetings will hereafter be held in the Carnegie Library.

---

DURING the month of June 316 emigrants were barred from entering the country owing to the fact that they were either diseased or physically deformed.

FOLLOWING the approaching medical congress at Madrid, there will be a conference of the members of the medical press, who are planning a national congress of deontology.

---

SINCE the outbreak of cholera in the Philippines there have been 21,408 cases and 16,105 deaths in the archipelago. Of this number 48 Americans and 18 Europeans have died since the epidemic began.

---

WORD has been received of the death of ex-Professor Schenk, who has lived in retirement since he was asked to resign from the Vienna faculty on account of his unscientific promulgation of his theory in regard to sex determination.

---

THE sixth annual meeting of the French Urological Society will be held at Paris, October 23 to 25. The principal question to be discussed is "Indications and Results of Nephrectomy." For further particulars address Dr. E. Desnos, 31 Rue de Rome, Paris.

---

SEVERAL recent graduates in medicine may secure interneships in the Craig Colony for Epileptics, at Sonyea, in Livingston county, N. Y. The positions are unpaid, but those who hold them get board, lodging, and laundry free. Further information may be had from Dr. W. P. Spratling, Sonyea, N. Y.

---

WHEN he is first engaged, a Russian surgeon starts upon the salary of $400 a year. On this pay he remains four years, when he is advanced to $500 yearly for four years. Four years later this is increased to $650; then to $750; the final remuneration, the highest which he can obtain, being $2,750 a year. When compared with the salaries of American army surgeons, it will be noted that Russia pays less than half the amount received by United States military surgeons.—*Exchange.*

THE French have a custom of sending a letter to their acquaintances on the death of a member of the family, with the full name, titles, dates, etc., of the deceased. One of these letters "*de faire part*" has been received by the friends of Dr. Felix Marsan, ex-interne of the hospitals of Paris, formerly of Martinique. It contains the death notice of eighty-five members of his family who perished in the Martinique catastrophe, parents, grandparents, brothers, sisters, uncles, aunts and cousins.—*Journal of the American Medical Association.*

---

FOUR-HUNDRED-DOLLAR PRIZE.—Dr. J. B. Mattison, Medical Director, Brooklyn Home for Narcotic Inebriates, offers a prize of $400 for the best paper on the subject: "Does the Habitual Subdermic use of Morphia Cause Organic Disease? If so, What?" Contest to be open two years from December 1, 1901, to any physician, in any language. Award to be determined by a committee: Dr. T. D. Crothers, Hartford, Conn., editor *Journal of Inebriety*, chairman; Dr. J. M. Van Cott, Professor of Pathology, Long Island College Hospital, Brooklyn, and Dr. Wharton Sinkler, Neurologist to the State Asylum for the Chronic Insane, Philadelphia. All papers to be in the hands of the chairman, by or before December 1, 1903, to become the property of the American Association for the study and cure of inebriety, and to be published in such journals as the committee may select.

---

LIEUTENANT JERE B. CLAYTON, assistant surgeon, U. S. A., in command of the medical expedition sent on the U. S. S. *Dixie* to relieve the sufferers on the island of Martinique, has transmitted his formal report to the surgeon-general of the army. Lieutenant Clayton says that as near as could be ascertained the cause of death was the explosion of an inflammable gas, which was emitted by the mountain. The most plausible explanation of the conditions found, he says, was given by Lieutenant Reilly, who suggested that the gas, as emitted by the mountain, was not inflammable until mixed with a certain quantity of oxygen, which mixture was reached at the time the gas arrived at St. Pierre. It was firmly asserted by all the survivors that every one in St. Pierre was dead three minutes after the explosion took place. The volcano on St. Vincent known as La Soufriere was also visited, and the cause of death there seems to have been suffocation by sulphur dioxide, or some similar gas. It is estimated that about two thousand deaths were caused by the eruption of La Soufriere.—*New York Medical Journal.*

---

TRI-STATE MEDICAL SOCIETY OF ALABAMA, GEORGIA AND TENNESSEE.—The fourteenth annual meeting of this popular organization will be held in Birmingham, Ala., Tuesday, Wednesday and Thursday, October 7, 8 and 9, 1902. All are invited to attend.

Reduced rates on the certificate plan have been granted by the Southeastern Passenger Association from all points in its territory (south of the Ohio and east of the Mississippi rivers). The following is a partial list of papers:

"President's Address." J. C. LeGrand, Birmingham.

"Adenoids." S. L. Ledbetter, Birmingham.

"Trachoma in Our Asylums and Hospitals." W. Cheatham, Louisville.

"An Analysis of One Hundred Cases of Refraction with Special Reference to Headache." A. W. Stirling, Atlanta.

"Purulent Ophthalmia." Frank Trester Smith, Chattanooga.

"Mastoiditis." L. G. Woodson, Birmingham.

"Indications for Operation in Mastoid Empyema." Samuel Kirkpatrick, Selma.

"So-called Membranous Croup." C. H. Peete, Macon, Ga.

"Anesthesia." G. W. J. Peters, Birmingham.

"Compound Fracture of the Skull." W. E. Wilder, Birmingham.

"Localization in Fracture of the Skull." G. Manning Ellis, Chattanooga.

"Gun-shot Wounds of the Abdomen with Report of Cases." R. E. Fort, Nashville.

"Report of Cases." G. A. Baxter, Chattanooga.

"Differential Diagnosis and Treatment of Hip Disease." W. F. Berry, Birmingham.

Gun-shot Wounds of the Liver." Wm. W. Harper, Selma, Ala.

"A Case of Septicemia, Laparotomy, Described by the Patient Himself." J. C. Newman, Helenwood, Tenn.

"Gastropaxy." H. Berlin, Chattanooga.

"Hernia." C. Holtzclaw, Chattanooga.

"Treatment of Hernia." German P. Haymore, Chattanooga.

"Treatment of Peritonitis." W. E. B. Davis, Birmingham.

"Appendicitis." W. A. Sellers, Birmingham.

"Appendicitis: Responsibility for Delayed Operation." W. F. Rochelle, Jackson, Tenn.

"Some Newer Methods of Hemoglobin Estimation." J. M. Mason, Birmingham.

"Leucocyte Count in Appendicitis." James Brew, Nashville.

"Report of a Case of Meningitis." D. S. Middleton, Rising Fawn, Ga.

"Puerperal Infection." T. A. Casey, North Birmingham.

"Puerperal Sepsis." J. C. Abernathy, Birmingham.

"Puerperal Sepsis." E. B. Ward, Selma.

"Placenta Previa, with Report of Case." C. W. Hilliard, Elba, Ala.

"Conservatism in Pelvic Work." W. G. Bogart, Chattanooga.

"Obesity and Senility in Relation to Accidental Injuries." W. L. Gahagan, New York.

"The Heroin Habit: Another Curse." Geo. E. Pettey, Memphis.

"The Value of State Sociological Societies in the Education and Development of the Race." R. R. Kime, Atlanta.

"Dynamic Sociology." Prof. Joel DuBose, Birmingham.

"Physiology of Education." J. W. McQuillan, Chattanooga.

"Some Further Remarks on the Medical Supervision of the Education of Boys and Girls." D. Y. Wilkinson, Montevallo, Ala.

"The Negro as a Criminal and His Influence on the White Race." Louis Edelman, Huntsville, Ala.

"Crime and Its Causes." H. L. Appleton, Centre, Ala.

"Crime: Its Causes and Evolution." Walter Lenehan, Nashville.

"Mind." R. C. Bankston, Birmingham.

"The Lawyer and the Doctor." Hon. Escar Floyd, Birmingham.

"The Doctor and the Lawyer: The Two Professions as Factors in Shaping Events to Higher and Better Attainment." Hon. John Tomlinson, Birmingham.

"The Relations between the Physician and the Apothecary." E. N. Russell, Birmingham,

"Medical Discoveries." W. L. Nolen, Chattanooga.

"Treatment of Gonorrhea." J. W. Johnson, Chattanooga.

"The Successful Treatment of Gonorrhea and Inflammatory Conditions of the Urethra by Packing with Antiseptic Oil Dressings. S. T. Rucker, Chattanooga.

"The Diagnostic Value of the Cystoscope." James E. Dedman, Birmingham.

"Tuberculosis." Y. L. Abernathy, Chattanooga.

"Tuberculosis." George Brown, Atlanta.

"Tuberculosis in the Negro." Searle Harris, Union Springs, Ala.

"Veratrum Viride and Its Indications." W. A. Barrett, East Lake, Ala.

"Gelsemium." Leroy Burdeshaw, Headland, Ala.

"Case of Bright's Disease with Valvular Heart Lesions, Benefited by Apocynum Cannabinum." R. B. Paine, Mandeville, La.

"Unilateral Sweating." E. Bates Block, Atlanta.

"Tetanus: Report of a Case." Frank B. Reagor, Shelbyville, Tenn.

"Diabetes Mellitus." Geo. R. West, Chattanooga.

"Cases Illustrating the Correction of Deformity by Operation Due to Infantile Paralysis." Michael Hoke, Atlanta.

"Curettage: Indications and Technique." Monroe Smith, Atlanta.

"A Study of Fifty-five Fatal Cases of Pertussus." Marian McH. Hull, Atlanta.

"Rabies: Its Nature and Preventive Treatment." J. N. Brawner, Atlanta.

"The X-Ray as a Therapeutic Measure." John W. Daniel, Savannah.

"A Duty of the Obstetrician." W. L. Champion, Atlanta.

Papers by Geo. S. Brown, E. M. Robinson, F. A. Lupton, B. L. Wyman, Wyatt Heflin, D. F. Talley, E. P. Riggs, B. G. Copeland, U. G. Peters, C. Lull, R. M. Cunningham, of Birmingham; L. L. Hill, Montgomery; W. F. Westmoreland, W. S. Elkin, J. M. Crawford, E. C. Davis, Atlanta; J. L. Hiers, Savannah.

